# Burden of frailty in the elderly population: perspectives for a public health challenge

**DOI:** 10.1186/s13690-015-0068-x

**Published:** 2015-04-10

**Authors:** Fanny Buckinx, Yves Rolland, Jean-Yves Reginster, Céline Ricour, Jean Petermans, Olivier Bruyère

**Affiliations:** Department of Public Health, Epidemiology and Health Economics, University of Liège, Avenue de l’Hôpital 3 – CHUB23, 4000 Liège, Belgium; Support Unit in Epidemiology and Biostatistics, University of Liège, Liège, Belgium; Gérontopôle, Toulouse, France; Bone and cartilage Metabolism Unit, CHU of Liège, Liège, Belgium; Geriatric Department, CHU Liège, Liège, Belgium; Department of Motricity Sciences, University of Liège, Liège, Belgium

**Keywords:** Frailty, Elderly, Public health, Prevalence

## Abstract

Frailty is a major health condition associated with ageing. Although the concept is almost universally accepted, its operational definition remains controversial. Anyway, this geriatric condition represents a huge potential public health issue at both the patient and the societal levels because of its multiple clinical, societal consequences and its dynamic nature. Here, we review existing definitions and assessment tools for frailty, we highlight consequences of this geriatric condition and we discuss the importance of its screening and prevention to limit its public health burden.

## Background

Frailty is a state of increased vulnerability to poor resolution of homoeostasis after a stressor event and increases the risk of adverse outcomes, including falls, delirium, and disability [1,2]. Frailty is the consequence of accumulated age-related defects in different physiological systems [3]. According to the World Health Organization, the global population of elderly people aged 60 years or more was 600 million in 2000; it is expected to rise to around 2 billion by 2050 [4]. With an aging population, there is a growing interest for frailty [5]. Indeed, a quarter to a half of people older than 85 years are estimated to be frail [1]. However, frailty remains an evolving concept lacking both a unique definition and diagnostic criteria to be used in clinical practice and epidemiological researches [6,7]. While researchers, policy makers and health care providers generally agree that frailty can have an important impact on affected individuals, their families, the health care system and the society, the concept of frailty remains controversial [5]. From a clinical perspective, frailty is crucial because it constitutes a condition of greater risk of adverse health outcomes, such as falls, hospitalization, disability and death [8]. Frailty is important from a societal perspective because it identifies groups of people in need of extra medical attention and at risk of high dependency. Frailty is also on concern when considering financial health care planning to better select management and prevention programs. Finally, as suggested in some recent studies, the frailty status might be reversible with the implementation of specific exercises programs [9-11] and nutritional supplementation [12,13]. Therefore identify frail elderly subjects is essential.

**Figure 1:**
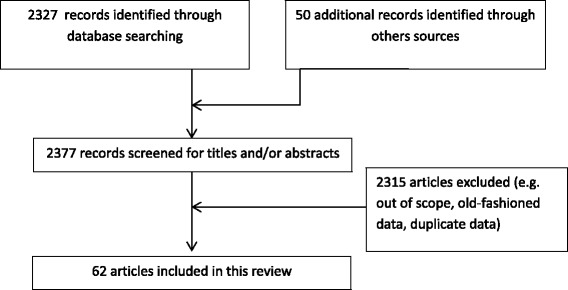
**Flowchart of litterature search.** Flowchart showing study selection for this review of the literature on frailty among elderly.

This work was not intended to be a systematic review but only to be a thematic one conducted by epidemiologists and geriatricians. Therefore, the purpose of this study was to review the recent literature on the definition of frailty, the burden of the disease and the challenges for public health (i.e. screening and prevention). To select the most recent articles, we carried out a search in the electronic database MEDLINE to identify studies published within the last 20 years. We also limited our search to articles about human frailty, written in English or French and concerning people aged over 65 years. The mesh term « frailty » was used in this research and to refine the search, the term “definition”, “prevalence”, “epidemiology”, “screening”, “consequences” and “intervention” were combined with “frailty” using Boolean indicators. Additional studies were identified by a manual search of bibliographic references of selected articles and existing reviews. More than 2300 articles were found. The most interesting references were selected on the basis of the previously reported goals (Figure 1).

## Main text

### Controversies in frailty definition

Frailty is a concept that has been used in clinical and research fields for more than two decades [14]. It is usually described as a clinical state of increased vulnerability to poor resolution of homoeostasis after a stressor event which increases the risk of adverse outcomes, including falls, delirium, and disability [1,2,8]. The European Union has placed specific importance on defining precisely frailty because, from an economic point of view, frail persons are high users of community resources such as hospitalization and institutionalization [2]. It is important to note that all frail elderly subjects do not experience the same symptoms and consequences and that frailty is not related to specific diseases, but is rather more present as a combination of consequences of co-morbidity [8]. In the last few years, frailty has been acknowledged to be not only a biological or physiological problem, but mainly a multidimensional concept [15]. Recently, an integral conceptual model of frailty reflecting part of the current thinking on frailty was established. This model uses the following definition which illustrates well this new concept: “Frailty is a dynamic state affecting an individual who experiences losses in one or more domains of human functioning (physical, psychological, and social), which is caused by the influence of a range of variables and which increases the risk of adverse outcomes [16]”. This definition reflects the changeability of frailty over time and emphasizes that the interacting factors in the physical, psychological and social domains are part of a complex dynamic system [15]. Moreover, some recent works consider cognition in the definition of frailty [1,17]. Based on an international consensus, “cognitive frailty” is an heterogeneous clinical manifestation characterized by simultaneous presence of both physical frailty and cognitive impairments [18]. The definition implies that cognitive frailty is characterized by reduced cognitive reserves, but is different from the physiological brain aging [19].

Thus, no consensus about the definition of frailty emerges from the literature. Despite significant work over the past decades, the debate continues between normal ageing on one hand, and pathophysiological entity on the other [14]. The choice of which components have to be included in the frailty definition continues to be a contentious issue with important implications. For example, although some authors have included disability and functional decline as components of frailty, others consider disability and functional decline as outcomes [20]. Moreover, within each of the physical, psychological and social dimensions, various determinants for frailty exist. Consequently, numerous assessment tools of frailty have been developed, generally on the basis of one or the other of these definitions. Most tools include a pre-frailty state allowing the identification of a subset of subjects at high risk of progressing to frailty [21]. These tools are grouped into three categories: subjective (self-report only), objective (inclusion of only directly measured components) or subjective and objective combined (mixed) [22].

### Assessment tools to identify frailty

Several frailty scales have been proposed on the basis of different conceptual models of frailty and two approaches have become popular [21]. The first model, or deficit model, consists of adding together individual’s number of impairments and conditions to create a Frailty Index [23]. This model consider frailty as a multidimensional risk state that can be measured more by the quantity than by the nature of health problems [24]. The second model, originally, defined a specific physical phenotype of frailty. This model views frailty as a biological syndrome resulting from cumulative decline across multiple physiological systems [24]. Other operational concepts of frailty can be considered on a spectrum between these two approaches [15,25,26]. According to Cesari [19], it is inappropriate to consider the frailty phenotype and the frailty index as alternatives or substitutable models. These two instruments are different and should rather be considered as complementary.

To our current knowledge, the instruments described below are frequently cited in the literature as validated tools to measure frailty.

- Frailty phenotype: Fried [8] defines a phenotype of frailty by the presence of three or more of the following components: shrinking, weakness, poor endurance and energy, slowness and low physical activity level. Presence of one or two deficits indicates a prefrail condition, while the absence of deficit indicates a robust state.
- Strawbridge questionnaire: The questionnaire developed by Strawbridge in 1998 [27], defines frailty as difficulty in two or more functional domains (physical, cognitive, sensory, and nutritive).
- Edmonton Frail Scale (EFS): The EFS samples 8 domains (Cognitive impairment, health attitudes, social support, medication use, nutrition, mood, continence and functional abilities). The maximum score is 17 and represents the highest level of frailty [28]. A score range between 0 and 3 defines a robust state, a score of 4 or 5 corresponds to the slightly frail state, a score range between 6 and 8 corresponds to the moderately frail state and a score range between 9 and 17 corresponds to the severely frail state.
- Clinical Frailty Scale (CFS): CSF is based on a clinical evaluation in the domains of mobility, energy, physical activity and function. The scale uses descriptors, icons and figures to stratify older adults according to their level of vulnerability and the score ranges from 1 (robust health) to 7 (complete functional dependence on others) [17].
- FRAIL Scale: The Frail Scale includes 5 components and considers deficits accumulated in these 5 domains, forming its acronym: Fatigue, Resistance, Ambulation, Illness, and Loss of weight. Frail scale scores range from 0–5 (i.e., 1 point for each component; 0 = best to 5 = worst) and represent frail (3–5), pre-frail (1–2), and robust (0) health status [29,30].
- Groningen Frailty Indicator (GgugFI) : The GFI consists of 15 self-report items and screens for loss of functions and resources in four domains: physical, cognitive, social, and psychological. Scores range from zero (not frail) to fifteen (very frail). A score of GFI of 4 or higher is regarded as frail [31].
- Share Frailty Instrument (Share-FI) : Using the five SHARE frailty variables (fatigue, loss of appetite, grip strength, functional difficulties and physical activity), DFactor scores (DFS) were determined using the SHARE-FI formula and based on the DFS value, the subject could then be categorized as non-frail, pre-frail, or frail [32].
- Tilburg Frailty Indicator (TFI): The TFI consists of 2 parts. Part A contains 10 questions on determinants of frailty and diseases (multi-morbidity); part B contains 3 domains of frailty (quality of life, disability, and healthcare utilization) with a total of 15 questions on components of frailty. The cut off point for frailty is defined as 5 points [33].
- Frailty index: The index is often expressed as a ratio of deficits present to the total number of deficits considered. It shows a consistent, sub-maximal limit at about 2/3 of the deficits that are considered. Frailty index includes 40 variables [34].
- The Gérontopôle Frailty Screening Tool [35]: Two different parts compose the instrument that has been developed as a screening tool. The first one appears as a questionnaire. Its main objective is to attract the general practitioner’s attention to very general signs and/or symptoms potentially indicating the presence of an underlying frailty status. In the second part, the general practitioner expresses his/her own view about the frailty status of the individual.

Specific tools are normally designed for specific purposes. Consequently, when choosing a tool, one should keep in mind the purpose for which the tool was originally designed [19].

### Epidemiology of frailty

Frailty is very common in older people. According to various studies, the prevalence of frailty in community-dwelling elderly adults varies from 4.0% to 59.1% [21], seems to increase with age, appears to be greater in women than in men and is more prevalent in people, with lower education and income, with poorer health and higher rates of comorbid chronic disease and disability. Indeed, Collard’s meta-analysis [21] shows that the average prevalence of frailty was statistically significantly higher in women (9.6%, 95% CI: 9.2-10%) than in men (5.2%, 95% CI: 4.9-5.5%) (p < .001). Another study in 2010 [36] shows convergent results since the prevalence of frailty was higher in women (60.1%) than in men (40.4%), (p < .001). This article also highlights that the prevalence increases with each 5-year age group before reaching a “stable value” (15.3% among 65–69 years; 18.6% among 70–74 years; 23.5% among 75–79 years; 22.4% among 80–84 years; 20.2% over 85 years). Fried [8] also notes that prevalence of frailty increases with each 5-year age group (3.2% among 65–70 years; 5.3% among 71–74 years; 9.5% among 75–79 years; 16.3% among 80–85 years; 25.7% among 86–90 years; 23% over 90 years). Furthermore, a recent survey of 7510 community-dwelling older adults in 10 European countries found that prevalence of frailty was higher in southern than in northern Europe which is consistent with an unexplained north–south health risk gradient [37]. African Americans are more likely to be frail than Caucasians [3,38]. Prevalence of frailty seems higher among nursing home residents than in community dwelling people with a general pooled prevalence of 10.7% [21], and more specific values of 34.9% in a Polish cohort [39], 48% in a Canadian cohort [40], and 68% in a Spanish cohort [41]. This could be explained because institutionalization could be a consequence of frailty [42]. Finally, several studies show that the prevalence of pre-frailty is around 47% [37,43,44] but no consensus exists about the prevalence rates of frailty [8,21,45,46]. The various definitions of frailty could partly explain this discrepancies [47]. Indeed, the difference between weighted rates of frailty according to physical phenotype (9.9%) versus broad phenotype (13.6%) was statistically significant [21].

Frailty is a dynamic process that can improve or worsen over time [1,2] but worsening is more common than improvement and the development of frailty frequently results in a spiral of decline that leads not only to an increased frailty status but also to a worsening disability, falls, admission to hospital and even death. Epidemiological data on transitions between frailty states (i.e. non-frail, pre-frail, and frail) were first reported by Gill et al. in a 4.5-year longitudinal study of 754 community-living adults aged 70 years and above [25]. During the follow-up, 58% of participants had at least one transition between any two of the three frailty states.

### Screening frailty

Many instruments for evaluating frailty have been developed in recent years. While the concept of frailty is now accepted by all, the introduction of screening in medical practice remains controversial [1] and operational criteria vary [24]. The choice of the screening tool for frailty has to be based on the definition which will best suit the needs of the researchers, clinicians or policy-makers [20]. Around the world, many initiatives are setting up screening for frailty and its management.

For example, a French national initiative, the HAS [48], suggests implementing a screening for frailty among people over 70 years old using the questionnaire elaborated by the « Gérontopôle in Toulouse » for the identification of frailty in primary care. Also, another French initiative, the PAERPA [49], concerns people over 75 years old and aims to identify and to prevent the risk of frailty. In Japan, a screening approach is being carried out widely using the Kihon checklist developed by the Japanese Ministry of Health [50].

To successfully combat frailty, we must implement the screening and management of frailty into clinical practice worldwide. From a public health point of view, the objective of screening frailty would ultimately reduce overall costs by reducing the rate of institutionalization and hospitalization [51]. Reducing the severity of frailty will provide large benefits for individuals, their families and for the society. Frail elderly subjects receiving care to counteract frailty are more susceptible to have less cognitive or functional decline, to present lower mortality rates and to experience fewer falls.

### Consequences of frailty

The frailty syndrome which makes individuals more vulnerable to adverse health outcomes through generally subtle and progressive physical changes, has attracted the attention of the medical and scientific communities as well as the public health department of numerous countries [52].

The association between frailty and adverse outcomes (falls, disability, hospitalization, care home admission and mortality) has been reported in four large cohort studies:

- The Cardiovascular Health Study (CHS) showed a predictive association between frailty and intermediate frailty status with incident falls, worsened mobility or activities of daily living (ADL) disability, incident hospitalization and death over 3 or 7 years of follow up, with hazard ratio ranging respectively from 1.82 to 4.46 and from 1.28 to 2.10 for the frail and intermediate groups [8].
- The Canadian Study of Health and Aging (CSHA) highlighted that increasing frailty was associated with an increased 5-year risk for death, with an odds ratio of 4.82 (95% CI: 3.74 - 6.21) among mildly frail people and 7.34 (95% CI 4.73- 11.38) among severely frail people. Moreover, in this study, frailty was the most important predictor of death and institutionalization (Odds ratio: 7.28 (95% CI 5.01-10.58) among mildly frail people and 8.64 (95% CI 4.92-15.17) among severely frail people) [53]. This study therefore shows that the risk for adverse health outcomes increased markedly with frailty and these risks persist after adjustments for age, sex, comorbid conditions, and poor self-rated health.
- The Women’s Health and Aging Study (WHAS) showed, in agreement with analyses in the CHS, that frailty strongly predicted all considered outcomes except falls and first hospitalization. Indeed, compared to robust individuals, frail women had a 6-fold higher risk of death and a more than 10-fold higher risk of incident instrumental ADL (IADL) and ADL disability and nursing home entry [54].
- The Study of Osteoporotic Fracture (SOF) showed that frailty was associated with increased odds of 2 or more falls in the subsequent year. Compared with robust women, women in the intermediate group had a 1.2- to 1.4-fold age-adjusted increase in risk (P < .04) and frail women had a 2.4-fold increase in risk (P < .001). Then, the odds of incident disability (≥1 new IADL impairment) were greater with increasing evidence of frailty. Compared with robust women, women in the intermediate group had an age-adjusted 1.8- to 1.9-fold increase in risk of disability (P < .001) and frail women had a 2.2- to 2.9-fold increase in risk of disability (P < .001). All-cause mortality rates were also higher with increasing evidence of frailty. Compared with robust women, women in the intermediate group had an age-adjusted 1.4- to 1.5-fold increased risk of death (P < .001) and frail women had a 2.4- to 2.7-fold increased risk of death (P < .001) [55].

Frail subjects are also at risk of iatrogenic disability which is defined as the avoidable dependence which often occurs during the course of care [56].

### The way ahead

Reducing the severity of frailty is supposed to provide large benefits for individuals, their families and for the society. Treating frailty in older people seems a realistic therapeutic and preventive goal. The lack of consensus regarding the definition and the components of frailty influences clinicians’ approach to intervention. Clinicians should specifically identify and target the dimensions of frailty identified with different assessment tools [57]. At this time, from the current published literature, four treatments appear to have potential to manage physical components of frailty: exercise, caloric and protein support, vitamin D and reduction of polypharmacy [2].

Firstly, exercise has been showed to improve outcomes of mobility and functional ability in two systematic reviews of home-based and group-based exercise interventions for frail elderly people [58,59]. Secondly, about the caloric and protein support, weight loss is known to be a major component of frailty [8], and caloric supplementations have enhanced weight gain and reduced mortality and complications in various studies [2]. Furthermore, some studies have suggested that protein supplementation could increase muscle mass, reduce complications, improve grip strength and produce weight gain [60-62]. Thirdly, vitamin D supplementation for elderly people who are deficient in the vitamin have reduced the number of falls and mortality even if the optimal modalities have not yet been defined [1,63,64]. Fourthly, polypharmacy is recognized as a possible major contributor to the pathogenesis of frailty [2]. Reducing medication could reduce costs and incidence of frailty among elderly subjects [65].

Frailty and disability could be successfully treated using an interdisciplinary multifaceted care program [66]. Van de Rest showed that resistance-type exercise training in combination with protein supplementation was beneficial in the cognitive domain [67]. Another study showed that multidisciplinary interventions, including exercises, nutritional and psychological management had a positive effect on various clinical outcomes for frail older people [57]. A systematic review showed that overall interdisciplinary interventions had a positive impact on residents’ outcomes in nursing home settings [68].

It is assumed that early interventions with frail or pre-frail people will improve quality of life and reduce costs of care. Several approaches have already been investigated in different clinical trials.

## Discussion

### Perspectives

Screening frailty and implementing early interventions could prevent the risk of loss of autonomy and the occurrence of adverse health events of people aged 65 or over, within 1 to 3 years [48]. In addition, the identification of frail individuals could help in improving the management of their comorbidities. Indeed, frail patients appear to have specific care needs, beyond care of underlying or coincident comorbidities and associated disability. Medical care for frail older adults needs to include ruling out, and treatment of, pathologic causes of progressive weakness, weight loss, decreased exercise tolerance, slowed task performance (i.e. walking speed), and/or low activity. Because frailty is a progressive condition that begins with a preclinical stage [8] it offers the possibility of early detection and thus of prevention.

It is admitted that frailty, because of the related adverse events, is costly for the patient and the society. However, few data exist on the potential financial gains of screening for frailty and there is no evidence on the economic implications of interventions targeting degree of frailty in the frail population. Identification of cost-effective interventions to reduce frailty may help health services to more efficiently allocate health care resources to those older people most at risk [69]. Identifying cost-effective means for reducing frailty has the potential to guide appropriate use of the limited resources available to improve outcomes in older people. Therefore, further data are needed and a cost-effectiveness study could fill the gap in the literature.

## Conclusions

Frailty has become a major health condition associated with ageing, and it contributes to many components of public health at both the patient and the societal levels. Although theoretical foundations of frailty are well established in the literature, and the concept almost universally accepted, a clear consensus on the definition of frailty does not emerge from the literature. Indeed, as this syndrome has been acknowledged to be a multidimensional concept, the choice of the components to be included in the frailty definition continues to be controversial. Whatever the definition or the assessment tool used, the burden of this syndrome and its costs for both the individual and the society are of concern in our ageing population. It seems thus essential for public health to implement the screening and multidisciplinary treatments of frailty.
